# Metabolic and Microbial Changes Associated With Diet and Obesity During Pregnancy: What Can We Learn From Animal Studies?

**DOI:** 10.3389/fcimb.2021.795924

**Published:** 2022-01-18

**Authors:** Caitlin Dreisbach, Hailey Morgan, Caroline Cochran, Adwoa Gyamfi, Wendy Ann Henderson, Stephanie Prescott

**Affiliations:** ^1^ Data Science Institute, Columbia University, New York, NY, United States; ^2^ College of Nursing, University of South Florida, Tampa, FL, United States; ^3^ School of Nursing, Columbia University, New York, NY, United States; ^4^ School of Medicine, University of Connecticut, Farmington, CT, United States; ^5^ School of Nursing, University of Connecticut, Storrs, CT, United States

**Keywords:** obesity, microbiota, pregnancy, metabolism, diet

## Abstract

The intestinal microbiota changes throughout pregnancy and influences maternal metabolic adaptations to support fetal growth. Obesity induces alterations to the microbiota that include decreased microbial diversity and shifts in microbial composition, though specific species changes are inconsistent between published studies. In animal models, probiotics and exercise moderate maternal weight gain and partially correct the maternal microbiota. Supplemental *Escherichia coli*, however, exacerbate maternal obesity during the perinatal period, lending weight to the theory that inflammation-induced gut epithelial barrier leak influences metabolic dysregulation. Although birth weight is not always altered when offspring are exposed to an obesogenic diet during gestation, insulin resistance and lipid metabolism are impacted through adulthood in association with this exposure and can lead to increased body weight in adulthood. Postnatal offspring growth is accelerated in response to maternal overnutrition during lactation. Offspring microbiota, metabolism, and behavior are altered in response to early exposure to high fat and high sucrose diets. Consequences to this exposure include impaired glucose and insulin homeostasis, fatty liver, and neurobehavioral deficits that can be ameliorated by improving the microbial environment. In this mini review, we provide an overview of the use of translational animal models to understand the mechanisms associated with changes to the gastrointestinal microbiota due to maternal obesity and the microbial impact on the metabolic changes of pregnancy.

## Introduction

Worldwide obesity rates have almost tripled in the last 35 years (World Health Organization (WHO), 2021). Nearly two billion adults over the age of 18 are overweight (B.M.I. 24-29 kg/m^2^), and 650 million are obese (B.M.I.> 30 kg/m^2^) (World Health Organization (WHO), 2021). Additionally, over 340 million children and adolescents aged 5-18 are overweight or obese (World Health Organization (WHO), 2021). These statistics contribute to the 2.6 million people who die each year due, in part, to having an overweight or obese BMI (World Health Organization (WHO), 2021). More specifically, maternal obesity, or obesity directly before or during pregnancy, is associated with an increased risk of complications during gestation, obesity, and neurodevelopmental abnormalities in offspring (Kim et al., 2017). The composition of the maternal intestinal microbiota changes throughout pregnancy and lactation as maternal metabolism adjusts to support the needs of the mother and the developing infant (Edwards et al., 2017). Obesity leads to altered microbial colonization, which may impact not only fecundity and pregnancy outcomes but the metabolic and developmental programming of offspring (Zhou and Xiao, 2018). While many studies focus on either human subjects or animal models, little work has been done to integrate the translational nature of these research questions.

Understanding the changes in the maternal microbiota because of obesity and overnutrition may elucidate the relationship between the microbiota and obesity-related adverse metabolic outcomes. Animal models have long been used to understand the mechanisms that may be involved in human pathophysiology as they 1) allow rigorous experimental control; 2) permit environmental, genetic, and experimental manipulation that is intolerable, unethical, or unsafe to humans, particularly when in vulnerable conditions such as pregnancy; and 3) facilitate molecular study at the tissue level of biologic phenomenon. Therefore, the purpose of this mini review is to synthesize recent research on the importance of the maternal microbiota to metabolic functioning during pregnancy, examine the influence of maternal obesity on offspring microbiota, and evaluate the potential of translational animal models to inform human clinical studies.

## Obesity and Maternal Microbiota During Gestation

Not surprisingly, because of its role in energy extraction, metabolism, hormone regulation, and inflammatory crosstalk, the maternal intestinal microbial community is altered soon after pregnancy begins and continues to change throughout gestation (Nuriel-Ohayon et al., 2016). The gut microbiome typically consists of several phyla including Bacteroidetes, Firmicutes, Actinobacteria, Proteobacteria, Fusobacteria, and Verrucomicrobia (Rinninella et al., 2019). Of these, Firmicutes and Bacteroidetes make up approximately 90% of the microbial environment within the gut (Rinninella et al., 2019). The relative abundance of specific microbes is often stable in individuals, barring changes as a result of disease processes and antibiotic use, and is influenced by an individual’s dietary habits, cultural lifestyles, physical environment, exercise habits, and age (Rinninella et al., 2019). During pregnancy, maternal fecal within-sample bacterial diversity (alpha diversity) decreases significantly throughout gestation. Maternal obesity reduces alpha diversity further, though species affected are different across studies. Notably, as women progress through pregnancy, they have dramatically expanded intestinal beta diversity (high levels of between-individual variation), which is globally distinct from early gestation or nonpregnant controls (Rinninella et al., 2019). This increase in intestinal beta diversity during the third trimester of pregnancy is largely independent of maternal health status, suggesting that it is driven by pregnancy itself (Rinninella et al., 2019). Proteobacteria and Actinobacteria are enriched in the third trimester and induce inflammation at the intestinal mucosal interface leading to increases in inflammatory cytokines, including TNFa (Rinninella et al., 2019). In a study examining the human maternal microbiome across pregnancy and into the postpartum period, researchers transferred third-trimester microbiota to germ-free mice, which resulted in increased inflammation, decreased glucose tolerance, and increased adiposity compared to germ-free mice colonized with first-trimester microbiota (Rinninella et al., 2019). High intestinal beta diversity persists into the postpartum period, which may impact infant colonization and energy harvesting capabilities.

During the gestational period, the high fat/high fat high sugar (HF/HFHS) diet has a temporal effect on microbial composition in animal studies. A study comparing mice on a HF diet to mice genetically predisposed to obesity (ob/ob) on a regular diet found a trend toward increased Firmicutes in the ob/ob mice, but a significant proportional increase in Firmicutes from baseline to 4-weeks in the HF diet group (*p* <.05) (Murphy et al., 2010). A significant proportional increase in Actinobacteria was seen in both the ob/ob and HF diet groups after the first four weeks (compared to baseline) but did not significantly increase in the following 4-week period (Murphy et al., 2010). Therefore, it appears that microbiota associated with genetically induced obesity flourish in the gastrointestinal tracts of wild type mice in the presence of HF diet, and these microbes persist or increase over time. While animal studies cannot be directly interpreted as relevant to humans because of different diets, habitats, and species adaptations, all the animals in the studies included in this review share similar microbial profiles to humans at the higher taxonomic levels except Felis catus, which are colonized almost entirely by Firmicutes on the lower fat, dry food diet and have a 25% relative abundance of Fusobacteria in the higher fat, higher carbohydrate, wet diet. Like humans, obese mouse, rat, and monkey dams have altered gut microbiota composition uniquely across studies compared to lean dams (Kirwan et al., 2002). Furthermore, humans, rats, mice, and sows have distinct microbial and metabolic signatures between pre-pregnancy, gestation, and lactation, strengthening translation of animal studies to human clinical practice (Zhou et al., 2019; Kimura et al., 2020; Zhou et al., 2020). Though most studies concentrate on the effects of maternal obesity and the obesogenic diet on the offspring microbiota and outcomes, several studies focus on the microbiota during gestation and lactation and the associated changes to the maternal metabolism in response to a HFHS diet, obesity, prebiotic supplementation, exercise, and non-caloric sweetener use.

## Obesity and Metabolism During Gestation

The basal metabolic rate in healthy pregnant women increases by 4%, 10%, and 24% during the first, second, and third trimesters, respectively, because of increases in tissue synthesis and body mass and increased cardiovascular, renal, and respiratory work effort (Butte, 2005). Glucose metabolism changes significantly throughout gestation. Fasting blood glucose levels and insulin sensitivity decrease as pregnancy progresses, even as hepatic glucose and insulin production increases, thus allowing euglycemia in the mother and glucose transport across the placenta to support fetal growth (Lain and Catalano, 2007). In late gestation, maternal insulin insensitivity is profound, allowing enhanced glucose availability and clearance by the placenta and the fetus (Sivan et al., 1999). In obese women, the basal glucose and insulin levels are further increased while hepatic glucose production remains unchanged, indicating an inability to regulate glucose production *via* insulin-dependent pathways in the liver. The physiologic mechanisms leading to insulin insensitivity during pregnancy are not well understood, but potential contributing pathways have been identified. Tumor necrosis factor alpha (TNFa), a cytokine and adipokine, is secreted from the placenta and is a candidate regulatory molecule in muscle and adipose tissue. TNFa promotes the phosphorylation of insulin receptor substrate 1, which interferes with insulin binding, leading to insulin insensitivity in muscle and adipose tissue (Kirwan et al., 2002). Maternal plasma-free fatty acid concentrations also play a role in reduced hepatic insulin sensitivity by downregulating insulin receptor signaling in hepatocytes, which allows gluconeogenesis and glycolysis to continue in the setting of elevated blood glucose (Hadden and McLaughlin, 2009). Increased insulin resistance and elevated free fatty acid concentrations lead to increased adipose tissue stores in healthy pregnant women providing an accessible calorie source for both mother and fetus and a reservoir of cytokines and inflammatory markers that contribute to dynamic maternal metabolism (Lain and Catalano, 2007).

Also noted is the length of high fat/high fat high sugar (HF/HFHS) nutritional exposure and its effect on metabolic processes over time. HF diet has been shown to induce marked glucose intolerance and compromised insulin response in mouse models after only one week (Winzell and Ahren, 2004). The longer the mouse is exposed to a HF diet, the more pronounced the effect on their metabolic function—increased weight, adiposity, and sustained hyperglycemia are typically seen within four weeks of the HF diet regimen, with further progression into obesity and related sequelae by 16 weeks (Wang and Liao, 2012). While the transition to pregnancy (first trimester) is marked by increases in ketone bodies, myo-inositol, butyrate, tryptophan, and phenylalanine, the transition from gestation to lactation is marked by decreases in branched-chain amino acids (BCAA) and urea cycle metabolites. Obese dams have decreased microbial abundance and poorer transitions between the different metabolic states. Physiologic insulin resistance seen in late gestation appears in early gestation in obese dams, contributing to increased maternal adiposity and insulin resistance at an earlier fetal developmental period (Paul et al., 2016; Paul et al., 2018). Obese metabolic profiles have altered fatty acid oxidation, amino acid metabolism, gut microbial metabolites, and phospholipid metabolism (Paul et al., 2016). Metabolites involved in glucose and fatty acid metabolism, methionine, and tryptophan metabolism to serotonin are altered in obese dams (Paul et al., 2018). However, while obese rat dams have higher weight gain during gestation and higher energy intake than lean rat dams on a standard diet (SD), both groups retained similar blood glucose and blood insulin levels during gestation (Paul et al., 2018). Maternal HF diet, however, also influences the methylation of genomic regions proximal to *PPARG* and *FGF-21* genes, pathways involved in fatty acid and glucose regulation (Ge et al., 2012; Wankhade et al., 2017). These changes in maternal metabolism may influence fetal metabolic programming.

Animal studies have examined the effect of prebiotics on microbial composition and metabolic functioning in obese dams. Compared to rat dams on an ad libitum HFHS diet, weight-managed or oligofructose (OFS)-supplemented dams have lower blood glucose and plasma insulin concentrations in response to an oral glucose tolerance test, lower fasting plasma leptin, and higher peptide-YY (PYY), a satiety hormone. Insulin sensitivity is improved during lactation across groups. Altering the microbiota by adding the prebiotic oligofructose (OFS) to the diet increases the relative abundance of the Actinobacteria, Bifidobacterium, which corrects some metabolic functions by increasing PYY and glucagon-like peptide-1 (GLP-1), satiety hormones which lead to normalized weight gain during gestation. Although there was no SD control group in this study, maternal HFHS diet dams without OFS have increased levels of precursors to ketone bodies, elevated metabolites involved in lipid metabolism, elevated branch chain amino acids, and elevated glucogenic amino acids compared to weight-managed dams also receiving the HFHS diet or those not weight-managed but supplemented with OFS (Paul et al., 2016). The ability to manipulate maternal metabolism, and possible fetal metabolic programming by means of altering the microbiota is a positive indication that such manipulations may be a possible avenue for human translational study.

## Offspring Microbiota and Metabolism

Maternal obesity, HF, or HFHS diets during pregnancy do not appear to consistently impact the initial birth weight of offspring, even though fatty acids and sugars pass freely to the fetus through the placenta, but typically do influence the weight of offspring over their lifetime (Smith et al., 1992). Metabolic programming of the offspring is also impacted, as offspring exposed to HF/HFHS diets during gestation display insulin resistance, glucose intolerance, and altered lipid profiles (Zhou et al., 2019; Kimura et al., 2020; Zhou et al., 2020). In a study examining the effects of germ-free (GF) and specific pathogen-free (SPF) environments on pregnant mice and their progeny, offspring from both GF and low-fiber fed dams expressed obesogenic phenotypes (increased weight gain and glucose intolerance) when exposed to HF diets during adulthood (Kimura et al., 2020). Interestingly, when dams in these conditions were treated with short-chain fatty acids (SCFA) during pregnancy, offspring were resistant to obesity *via* SCFA-specific axes when exposed to the same HF diet after weaning. SCFAs are essential to the normal development of metabolic and neural systems in the pre-and postnatal period, affecting the metabolic programming of offspring in the embryonic stage (Kimura et al., 2020). This supports the notion that a decrease in microbial organisms related to diet- or antibiotic-induced gut dysbiosis that produce these essential metabolites can promote susceptibility to metabolic dysfunction and obesity in offspring through disruption of normal postnatal metabolism.

Research examining the effect of maternal exercise during pregnancy has shown similar ameliorative effects related to SCFA production (Zhou et al., 2020). Compared to SD and HF non-exercise groups, pregnant mice fed HF diets with voluntary access to exercise wheel activity prior to and during pregnancy had improved insulin sensitivity and reduced metabolic dysfunction in offspring as they transitioned into adulthood (Zhou et al., 2020). This metabolic improvement was associated with the protection of SCFA-producing bacteria in the HF exercise group. These diets during lactation and initial microbial colonization impact growth as differences in body weight or body fat content are observed before weaning, in some instances, before the offspring are of age to trial solid foods (Fåk et al., 2012; Paul et al., 2016; Bruce-Keller et al., 2017; Paul et al., 2018). Offspring weaned to the HF/HFHS diet typically become obese over time, though there are potential sex differences noted in the literature. Germ-free mice colonized with the cecal microbiota from donor mice on a HF diet had significantly heavier female offspring than SD dam’s offspring before weaning and had higher body fat by nine weeks of age even though all offspring were weaned to a SD (Bruce-Keller et al., 2017). Offspring exposed to overnutrition during gestation and then weaned to a SD do not always weigh significantly more than offspring maintained on a SD but exhibit more adiposity, insulin resistance, glucose intolerance, and lipid profile disorders in adulthood (Ma et al., 2014; Buffington et al., 2016; Wankhade et al., 2017). Offspring exposed to SD during gestation then weaned to the HF diet do surpass the weights of those maintained on the SD, though it took more time to match the weights of those fed the HF diet throughout gestation, lactation, and after weaning (Wankhade et al., 2017).

Overnutrition during pregnancy leads to decreased alpha diversity in offspring and distinct clustering in principal coordinate analyses of beta diversity, though drivers of diversity are different between studies. Most of the differences in relative abundance in the microbiota of offspring in the special diet groups occur at the lower levels of taxonomic classification within the Firmicutes phylum. This is perhaps not surprising as this phylum, along with the phylum Bacteroidetes, usually dominates the intestines of mammals (Jandhyala et al., 2015). Firmicutes populations are expanded while populations from Proteobacteria, Actinobacteria, and Bacteroidetes are reduced in over-nourished offspring. Sex differences exist in both the microbiota composition and the obesity phenotype of offspring but were inconsistent between studies (Bruce-Keller et al., 2017; Dennison et al., 2017). A HF diet is also associated with physical changes in the gastrointestinal tracts of offspring. For example, the pH of the stomach is increased, the protein concentration of the duodenum is decreased, sucrase and maltase enzymatic activity is decreased, and there is increased intestinal permeability in over-nourished offspring (Fåk et al., 2012). Dams that receive the prebiotic OFS have increased abundance of the Actinobacteria : Bifidobacterium and the Firmicutes : Clostridium, with altered SCFA production; and they have offspring with increased satiety hormones, PYY and GLP, leading to reductions in body fat (Paul et al., 2016). Offspring on the HFHS diet have higher glucose but similar insulin levels, higher leptin levels, and lower ghrelin levels compared to those on the HF diet alone. Offspring on the HF diet have normal glucose and triglyceride levels but higher cholesterol and non-esterified fatty acid levels leading to increased liver steatosis and fibrosis (Paul et al., 2016; Dennison et al., 2017; Wankhade et al., 2017). Offspring exposed to the HF diet have increased inflammation and inflammatory markers haptoglobin in serum and *LY6D* in the liver (Fåk et al., 2012; Wankhade et al., 2017).

Mechanisms by which changes in the microbiota influence the offspring’s growth and development are investigated though the changes in microbial populations induced by gestational overnutrition are not the same between studies. Structural (intestinal hyperpermeability) and chemical (decreased stomach pH) changes occur in the gastrointestinal tract because of exposure to the HF diet, which may allow bacterial components or products to bypass the body’s normal defenses (Fåk et al., 2012). Increases in the bacterial components and metabolites promote upregulation of inflammatory signaling pathways leading to liver steatosis and fibrosis (Wankhade et al., 2017). Changes in glucose, insulin, and fatty acid metabolism during gestation and lactation alter placental energy transactions and the availability of substrates for neurotransmitter formation (Wankhade et al., 2017; Dennison et al., 2017). Thus, alterations in normal colonization result in increased obesity and metabolic dysfunction by many different mechanisms (Ma et al., 2014; Bermingham et al., 2018).

## Discussion

The information presented in this review reaffirms the importance of considering animal studies in translational research for obese pregnant individuals. Diversity changes associated with adaptations to normal pregnancy are intensified in the setting of maternal obesity and overnutrition (Paul et al., 2018; Zhou and Xiao, 2018). Differences in microbial colonization patterns during gestation because of diet are more visible in animals than in human studies, perhaps because of the controls on genetics, environment, sample collection, and diet consumption. Metabolic profiles shift throughout normal gestation and lactation because of shifts in the hormonal, inflammatory, and nutritional environment, resulting in increased weight gain, blood glucose and blood insulin levels, and free fatty acids. In the context of maternal obesity and overnutrition, metabolic profiles shift more erratically, exposing developing offspring to increased levels of glucose, insulin, leptin, and BCAA leading to altered placental energy transfer. Obesity-induced methionine deficiency may interfere with methylation pathways involved in normal fetal development. Additionally, decreased tryptophan to serotonin metabolism may impact neurodevelopment and behavior in offspring (Paul et al., 2018).

Some studies reported no significant differences in offspring birthweight between dams fed HF or SD; instead, they highlighted the more frequent presence of differences in metabolic programming between these offspring and their susceptibility to obesity in adulthood. This is interesting, given previous associations between high maternal BMI and babies born large for gestational age, and may reflect the importance of the timing of maternal obesity and overnutrition to impact fetal developmental programming, or the effects of maternal microbial ecology (Jones et al., 2009). Although there is some evidence that the fetus begins acquiring its microbiota even before birth (Perez-Muñoz et al., 2017), the process of acquiring a stable adult-like microbiota takes up to 3 years in humans (Wankhade et al., 2017). Like germ-free mice, the nearly sterile fetus may not have the microbiota necessary to respond to HF diet exposure until after birth and colonization with a microbiota skewed toward obesity. Perhaps the maternal microbiota composition affects fetal metabolic programming independent of nutrition. More research is needed on maternal microbiota composition on fetal developmental programming. The bacterial constituents important for inducing obesity are challenging to determine as transplanted microbes from both obese and lean mice can induce obesity in HF diet germ-free mice (Rabot et al., 2016). Accordingly, offspring weaned to SD maintain normal weights despite their pre-weaning diet while offspring weaned to HF diet have increased weights even if they had been exposed to SD before weaning and during gestation (Buffington et al., 2016; Wankhade et al., 2017). Mice never exposed to the HF diet, and only to the microbiota of HF-fed mice, have increased body weight and fat content in a sex-dependent manner (Bruce-Keller et al., 2017). Several studies report that there are responders and non-responders to HF diet feeding among genetically identical test subjects, indicating that not all bacterial communities interact with host genetics to produce the obese phenotype (Ma et al., 2014).

Diet changes elicit rapid shifts in gut microbial communities (David et al., 2014). As the fetus does not always respond to the maternal HF diet with increased body weight before birth but does respond before consuming food other than breastmilk, it appears that the diet induces changes to the maternal microbiota, which then shifts and induces metabolic alterations in the mother and the fetus. The lack of obesity when HF-exposed offspring are weaned to a standard diet, but continued glucose intolerance, insulin resistance, and serum fatty acid elevation point to the influence of the microbiota in fetal metabolic programming. Delayed onset of obesity when SD-exposed offspring are weaned to the HF diet and increased growth rates of offspring of germ-free mice when the pregnant dams are exposed to microbiota transplant indicate that the diet-induced changes to the microbiota during development have lasting metabolic impact on offspring. Exposure to the HF diet *in utero* may alter the offspring’s metabolic programming as changes in glucose tolerance, insulin resistance, lipid profiles, and satiety hormones are observed in adult offspring even though increased body weight may not be observed (Zhou et al., 2019; Zhou et al., 2020). The proposed mechanism by which the microbiota regulate offspring metabolic programming includes SCFA production and free fatty acid receptor signaling in the murine fetal sympathetic nervous system, adipose tissue, and pancreas that disrupts energy homeostasis (Kimura et al., 2020). Changes to the maternal microbiome through exercise, non-nutritive sweeteners, or fiber supplementation result in changes to the maternal microbial milieu and maternal and offspring metabolism (Zhou et al., 2019; Zhou et al., 2020). The occurrence of HF diet non-responders seems to indicate that there is also an interaction between host genetics and microbial inhabitants that influence the metabolic changes that lead to obesity. Fåk et al. (2012) explored this hypothesis by inducing increased obesity over HF-induced obesity by adding Proteobacteria and *Escherichia coli* to the drinking water of dams and offspring receiving a HF diet (Fåk et al., 2012).

Synthesis of results is challenging because of the different diets used, experimental species and conditions tested, taxonomic levels reported, and time points evaluated. However, variations between animal studies are fewer than those that occur between human studies because of the ability to exert stringent controls. There are important limitations to be considered when translating research in animal models to humans, including overarching biological differences between human and rodent subjects, housing and environmental conditions, and the ability to strictly control the diet in animal models to the degree that is irreplicable in human samples. Additionally, certain neuroendocrinological developmental processes occur at different times in the developing rodent versus human. The HPA axis and appetite regulatory network in rodents develops during the postnatal period, in contrast to developing during the third trimester in humans hindering comparisons between the stress response and eating behaviors (Rinaudo and Wang, 2012). Human pancreatic cells continue to go through remodeling until around age 4; in rats, the pancreas is developed later in pregnancy and experiences important remodeling around the time that offspring are weaned (Rinaudo and Wang, 2012). Data regarding comparisons in insulin production in the context of offspring of obese mothers should be evaluated with this fact in mind. As mentioned previously, in many animal studies, the methodology utilized would be unethical to replicate in human subjects, especially those who are pregnant. However, the ability to manipulate the nutrients, environment, genetics, and microbiota of animals, in addition to the ability to study the effects of changes *in vivo* and *in vitro*, is an important adjunct to human research to fully understand the mechanisms by which the microbiota interacts with the host to support the developing fetus and neonate for optimal health. Animal studies are also invaluable in closely evaluating the impact of obesity and overnutrition during gestation, lactation, and microbial colonization. The microbiota is an exciting target for influencing maternal and fetal metabolism, particularly as it is easily manipulated and may provide an option for therapy that confers a life-long benefit. Future studies should examine the moderating effect of probiotic supplementations and physical exercise in obese, pregnant dams and the microbial colonization, metabolic and neural outcomes in offspring over their lifetime.

## Conclusion

Studies across species reveal how changes in the maternal microbiota during pregnancy are necessary to support the normal physiologic changes during pregnancy and metabolic programming of offspring. Changes in maternal microbiota induced by various methods can lead to long-lasting changes in maternal and offspring metabolic health, with mechanisms such as molecular signaling by metabolites and inflammatory cytokines or the alteration in gut epithelium allowing bacterial invasion. More research is required to determine the mechanisms by which the intestinal microbes participate in or respond to changes in the gastrointestinal tract that result from maternal overnutrition in humans, but that they have a role in the outcomes associated with maternal overnutrition by altering the substrates available for maternal, placental, and fetal metabolic processes are evident.

## Author Contributions

CD and SP conceptualized the manuscript. SP and CC performed the search of the literature. CD and CC created Table 1 based on the search results. CD and SP drafted the manuscript with all authors (CC, AG, WH, HM) providing substantial revisions. All authors contributed to the article and approved the submitted version.

## Conflict of Interest

The authors declare that the research was conducted in the absence of any commercial or financial relationships that could be construed as a potential conflict of interest.

## Publisher’s Note

All claims expressed in this article are solely those of the authors and do not necessarily represent those of their affiliated organizations, or those of the publisher, the editors and the reviewers. Any product that may be evaluated in this article, or claim that may be made by its manufacturer, is not guaranteed or endorsed by the publisher.
